# Supplementary material for: A set of multi-entry identification keys to African frugivorous flies (Diptera, Tephritidae)
Source: Zookeys. 2014 Jul 24;(428):97–108. doi: 10.3897/zookeys.428.7366 (PMC4143993; doi:10.3897/zookeys.428.7366)
Supplement: Supplementary material 6 — Key to Ceratitis [file zookeys-428-097-s006.zip › SF6_ZooKeys_key to Ceratitis/key/SF6_ZooKeys_key to Ceratitis/Media/Html/Ceratitis antistictica .htm]

Ceratitis (Ceratalaspis) antistictica Bezzi


***Ceratitis*** **(*Ceratalaspis*)
*antistictica* Bezzi**

*Ceratitis stictica* var. *antistictica*
Bezzi, 1913: 20.

Body
length: 4.35 mm; wing length: 4.15 mm.

Female

(Based
on the original description, with additional notes based on the single BPBM
specimen).

Head.
Antenna yellow-orange. Third antennal segment twice as long as second segment.
Arista with short to medium long hairs over entire length (original description
mentions quite long hairs). Frons with short scattered hairs which are the same
colour as the frons; convex, in lateral view not distinctly projecting forwards
at antennal implant. Face yellow colour. Chaetotaxy normal for subgenus.

Thorax.
Postpronotum yellowish, more or less the same colour as centre of mesonotum
(slightly less orange); with small darkish spot. Ground colour of mesonotum
yellow-orange. Chaetotaxy normal for subgenus. Inner scapular setae pale, outer
reddish. One anepisternal bristle. Scutellum mainly yellow, sometimes basally
somewhat more whitish; with three black separate markings apically; basally two
dark distinct spots (not clear in BPBM specimen since damaged by insect pin).
Subscutellum pale with three dark separate spots except along dorsal edge where
touching.

Legs.
Yellow; setation typical for subgenus, mainly pale especially on femora.
Posterior and posterodorsal rows on fore femur pale. Ventral bristles on femora
yellow.

Wing.
Bands with markings extensively yellow. Banding, marginal band continuous;
cubital and medial bands free; discal band broad occupying most of basal part of
wing; crossvein r-m at middle of discal cell. Crossvein dm-cu posteriorly more
inwards than anteriorly.

Abdomen.
Yellow. Setation and banding typical for subgenus. Oviscape shorter than
abdominal terga 3-6 combined.

Male

No
material seen. The original description mentions no apparent differences.��

(Description
after De Meyer, 1998)
